# Autophagy in Its (Proper) Context: Molecular Basis, Biological Relevance, Pharmacological Modulation, and Lifestyle Medicine

**DOI:** 10.7150/ijbs.95122

**Published:** 2024-04-22

**Authors:** Miguel A Ortega, Oscar Fraile-Martinez, Diego de Leon-Oliva, Diego Liviu Boaru, Laura Lopez-Gonzalez, Cielo García-Montero, Miguel Angel Alvarez-Mon, Luis G Guijarro, Diego Torres-Carranza, Miguel A Saez, Raul Diaz-Pedrero, Agustin Albillos, Melchor Alvarez-Mon

**Affiliations:** 1 Department of Medicine and Medical Specialities, Faculty of Medicine and Health Sciences, University of Alcalá, 28801 Alcala de Henares, Spain.; 2 Ramón y Cajal Institute of Sanitary Research (IRYCIS), 28034 Madrid, Spain.; 3 Department of Surgery, Medical and Social Sciences, Faculty of Medicine and Health Sciences, University of Alcalá, 28801 Alcala de Henares, Spain.; 4 Unit of Biochemistry and Molecular Biology, Department of System Biology (CIBEREHD), University of Alcalá, 28801 Alcala de Henares, Spain; 5 Department of General and Digestive Surgery, Príncipe de Asturias Universitary Hospital, 28805 Alcala de Henares, Spain.; 6 Pathological Anatomy Service, Central University Hospital of Defence-UAH Madrid, 28801 Alcala de Henares, Spain.; 7 Immune System Diseases-Rheumatology, Oncology Service an Internal Medicine (CIBEREHD), Príncipe de Asturias University Hospital, 28806 Alcala de Henares, Spain.

**Keywords:** ATG proteins, autophagosome, aging, pharmacological modulation, lifestyle habits

## Abstract

Autophagy plays a critical role in maintaining cellular homeostasis and responding to various stress conditions by the degradation of intracellular components. In this narrative review, we provide a comprehensive overview of autophagy's cellular and molecular basis, biological significance, pharmacological modulation, and its relevance in lifestyle medicine. We delve into the intricate molecular mechanisms that govern autophagy, including macroautophagy, microautophagy and chaperone-mediated autophagy. Moreover, we highlight the biological significance of autophagy in aging, immunity, metabolism, apoptosis, tissue differentiation and systemic diseases, such as neurodegenerative or cardiovascular diseases and cancer. We also discuss the latest advancements in pharmacological modulation of autophagy and their potential implications in clinical settings. Finally, we explore the intimate connection between lifestyle factors and autophagy, emphasizing how nutrition, exercise, sleep patterns and environmental factors can significantly impact the autophagic process. The integration of lifestyle medicine into autophagy research opens new avenues for promoting health and longevity through personalized interventions.

## Introduction

Autophagy is a conserved cellular self-eating process that plays a critical role in maintaining cellular homeostasis and responding to stress. It involves the degradation and recycling of damaged components, organelles, and molecules to ensure an adequate function of the cell. The process of autophagy includes three main types: macroautophagy, microautophagy, and chaperone-mediated autophagy. Macroautophagy forms autophagosomes, double-membrane vesicles that engulf cytoplasmic components and fuse with lysosomes for degradation and recycling. In microautophagy, materials are directly taken up into lysosomes by invagination. Chaperone-mediated autophagy selectively transports proteins to lysosomes for degradation with the help of chaperones. Autophagy plays a crucial role in cell survival, proliferation, metabolism, senescence, modulation of host defenses, and various other processes that contribute to maintaining cell integrity and function 1. Because of that, a growing number of studies have identified autophagy as a major hallmark of health, but also of disease 2,3. In this context, autophagy modulation arises as a promising translational intervention for sustaining health, delaying aging and also to prevent or ameliorate the initiation and progression of an array of diseases 4,5. Two major strategies directed to regulate autophagy can be remarked: pharmacological agents, frequently directed to specific activators/inhibitors of autophagy 6 and lifestyle medicine, a growing area of research that include different strategies like diet, physical activity, sleep and other modifiable habits that also plays a major role in the activation/inhibition of autophagy 7.

This integrative narrative review aims to shed light on the role of autophagy in cellular homeostasis and its broader implications in diverse biological processes. By comprehensively exploring the cellular and molecular basis of autophagy, its importance in aging and disease, the broad pharmacological modulation and the impact of lifestyle factors, we hope to improve our understanding of autophagy and stimulate innovative research and clinical applications that harness the full potential of this process for human health and well-being.

## Cellular and molecular basis of autophagy

### Macroautophagy

**Figure 1:**
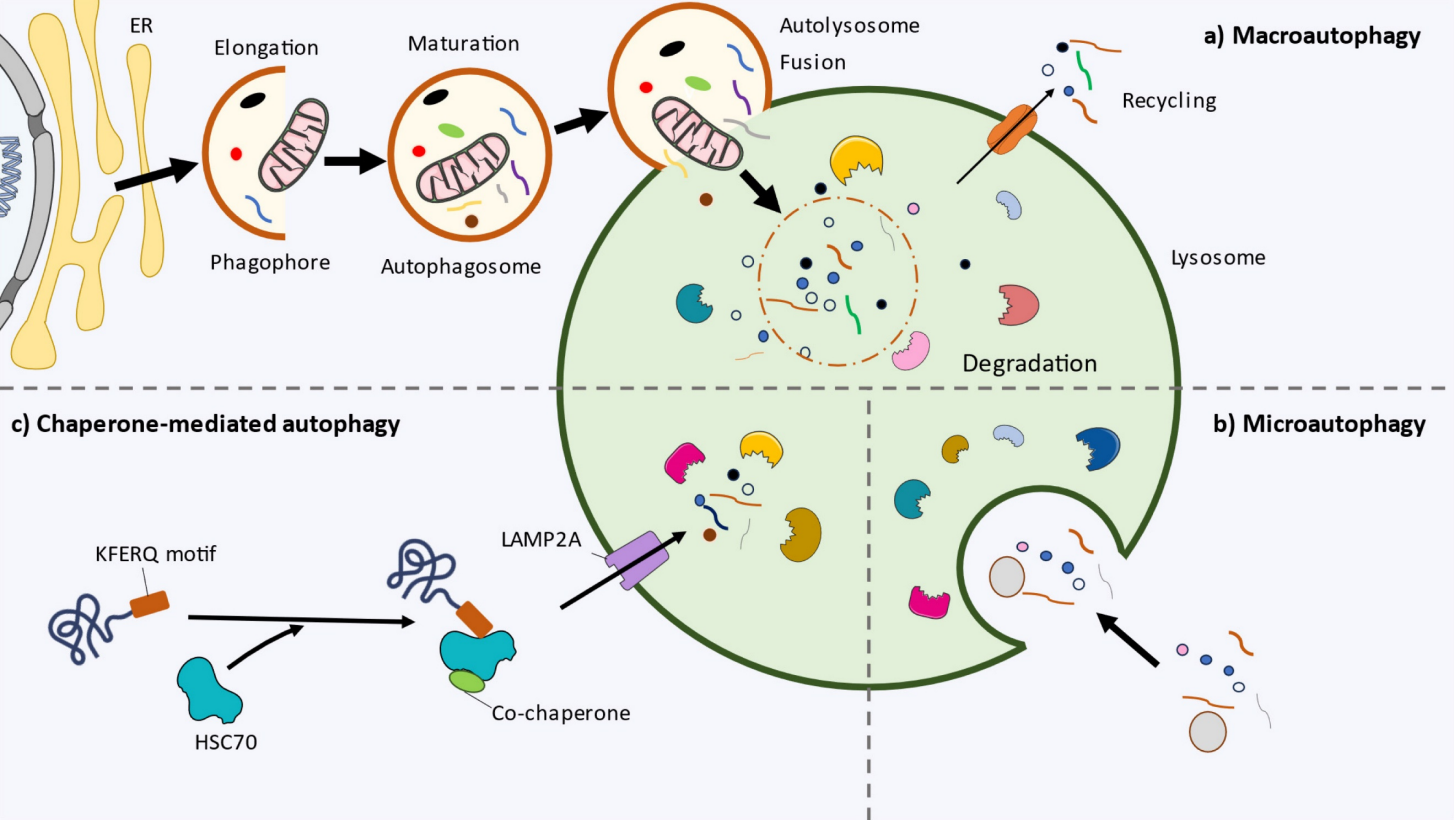
Cellular and molecular basis of autophagy. a) Macroautophagy. The initiation of autophagy and the nucleation of phagophore starts inside the endoplasmic reticulum (ER). During elongation, the phagophore engulfs the cytoplasmic materials. The phagophore closes forming the double-membrane vesicle autophagosome. Then, it fuses with the lysosome (autolysosome), where the inner autophagosomal membrane and cargo are degraded by the lysosomal hydrolases. The remaining catabolites are transported to cytoplasm and recycled by the cell. b) Microautophagy. The cytoplasmic material is captured through direct invagination of the lysosome. c) Chaperone-mediated autophagy (CMA). In multicellular organisms, proteins with the pentapeptide motif KFERQ are recognised and transported by the chaperone HSC70 and cochaperones to the lysosome. The LAMP2A receptor imports the content to the lysosome.

Macroautophagy, commonly referred to as autophagy, consists of the formation of an autophagosome, a double-membraned cytosolic vesicle, and the uptake of dysfunctional organelles and aggregated proteins leading to the degradation and recycling of these components after fusion with lysosomes (Figure 1a). In mammalian cells it develops in the following steps.

#### Initiation

The initiation phase involves the activation of autophagy in response to various internal and external stimuli, such as nutrient deprivation (amino acid starvation or glucose depletion), hypoxia, oxidative stress, accumulation of damaged organelles, physical exercise or pathogen invasion 8,9. In these contexts, the upstream regulators of autophagy mammalian target of rapamycin complex 1 (mTORC1) and AMP-activated protein kinase (AMPK) are inhibited and activated, respectively. Thus this leads to the assembly and activation of the ULK complex, composed of ULK1/2 (unc-51 like autophagy activating kinase 1/2; yeast Atg1), ATG 13, ATG 101 and FIP200 (focal adhesion kinase (FAK)-interacting protein of 200 kDa, also known as RBCC1) 10. Once activated, ULK1/2 phosphorylates itself and the ATG13 and FIP200 proteins, leading to the assembly of the ULK1/2 complex 11. This complex then localizes to a specific site in the cell called the phagophore assembly site (PAS, also referred to as preautophagosomal structure), which serves as a platform for autophagosome biogenesis. The current consensus states that upon activation the ULK complex translocates to a specific localization of endoplasmic reticulum (ER) marked by ATG9 12. Several mechanisms seem to be implicated in the recruitment of ULK1. They include the interaction of ULK1 with the gamma-aminobutyric acid receptor-associated protein (GABARAP), small GTPase RAB1A/ C9orf72 and ER contact proteins VAMP-associated protein A (VAPA) and VAPB 13.

Despite the similarity between ULK1 and ULK2 and the redundant or interchangeable role that had been assigned to them previously, there is growing evidence about their separate functions due to differences in autophagy‑related interactors and their post‑translational and transcriptional regulators 14. Furthermore, although it is well described the role of ULK1/2 complex in the initiation of autophagy, under certain conditions autophagy is initiated independently from it or mTOR, e.g.: acute hypoxia-induced autophagy or ammonia-induced autophagy 15,16.

mTOR, AMPK and p53 are the main upstream regulators of autophagy 17. Briefly, in basal conditions mTOR inhibits autophagy by phosphorylation of ULK1/2, ATG13 and the transcription factor EB (TFEB), which is in charge of the transcription of the autophagy machinery 18,19. During nutrient starvation, AMPK becomes activated and phosphorylates both ULK1/2, at additional sites to enhance its activation, and mTOR, specifically in the T-loop, resulting in mTOR inhibition. These events collectively trigger autophagy activation 20,21. The activation of the transcription factor p53 leads to the upregulation of ULK1/2, AMPK, or TSC2 through transcriptional processes. Conversely, p53 can also exert a negative regulatory effect on autophagy by inhibiting the expression of ULK1 and ATG7. Additionally, p53 can directly interact with BECN1, further modulating autophagy in response to various cellular conditions 22,23.

#### Nucleation

Activation of ULK1/2 complex leads to recruitment of class III phosphatidylinositol 3-kinase complex I (PI3KC3-C1), which consists of the lipid kinase PIK3C3/VPS34 (phosphatidylinositol 3-kinase catalytic subunit type 3), PIK3R4/VPS15/p150 (phosphoinositide-3-kinase regulatory subunit 4), BECN1/Beclin 1, ATG14/ATG14L and NRBF2 (nuclear receptor binding factor 2) 24. ULK1 phosphorylates the subunits of PI3K3C3-C1: PIK3C3 (S249), BECN1 (S15/30), ATG14 (S29) and PIK3R4 (S813/S861/S865/S879/S1039/S1289) 25-28. The phosphorylation of PIK3C3 facilitates its binding with GABARAP, GABARAPL1 (GABARAP-like 1), and LC3. Additionally, the phosphorylation of BECN1 and ATG14 enhances the lipid kinase activity of the complex. Together, these coordinated phosphorylation events enable the precise initiation of autophagy. The translocation of the PI3KC3-C1 is mediated by the subunit ATG14, essential for autophagy 29.

PIK3C3 is responsible for generating phosphatidylinositol 3-phosphate (PI3P), a key signaling lipid, at a cup-shaped ER subdomain known as the omegasome. It phosphorylates phosphatidylinositol at the 3' position on the inositol ring 30. PI3P and PIK3C3 also have significant roles in other cellular processes, including endocytic trafficking, phagocytosis, cytokinesis, and nutrient sensing 31. Inside the omegasomes, the phagophores (also known as isolation membranes) nucleate, elongate and engulf the cargo, eventually forming the autophagosome which dissociates from the ER 32,33. The curvature of the omegasome appears to facilitate the exposure of PI3P, resulting in the recruitment of PI3P-binding proteins, particularly double FYVE containing protein 1 (DFCP1) and WIPI/PROPPIN (WD-repeat protein interacting with phosphoinositides) family 34. DFCP1 is an ER-localized protein that relocates to the omegasome upon autophagy induction, but its precise function remains poorly understood. It is commonly used as a marker for the omegasome, and recent research indicates its involvement in lipid droplet formation within the ER 35. Moreover, recent studies have revealed that DFCP1 possesses ATPase activity, which mediates the release of autophagosomes specifically in selective autophagy processes, but not in non-selective autophagy 36. On the other hand, mammals possess four WIPI proteins, WIPI1-4, each characterized by seven bladed β-propellers. These proteins act as essential effectors of autophagy by binding to PI3P through the FRRG motif, inserting into the membrane through a hydrophobic loop in blade six, and subsequently recruiting downstream ATG proteins 37,38. In nucleation step, isoforms WIPI2B and WIPI2D bind and recruit the ATG5-ATG12-ATG16L1 complex by interacting with ATG16L1 39,40. In this sense, Jensen et al. showed recently that the interaction between the ATG12-ATG5-ATG16L1 complex and the early phagophore rim may stabilize membrane curvature and promote the growth of the autophagosome 41. Interestingly, it seems that WIPI2 is recruited to the membrane by RAB11A, a protein of recycling endosomes, where PI3P is also formed upon starvation 42.

#### Elongation

During the elongation phase of autophagy, the phagophore undergoes expansion and elongation, a process facilitated by the recruitment of autophagy-related proteins and the lipidation of LC3, a ubiquitin-like protein. This crucial step enables the autophagosome to engulf cellular cargo for subsequent degradation and recycling. Key to the elongation phase is the lipidation of the microtubule-associated protein 1 light chain 3 (LC3), a member of the ATG8 family 43. LC3 is initially synthesized as an inactive precursor, which Arg C-terminus residues are removed by the cysteine protease ATG4. Then, ATG7 (E1-like activating enzyme), ATG3 (E2-like conjugating enzyme) and the supramolecular complex ATG12-ATG5-ATG16L1 (E3-like ligase complex) act subsequently to covalently link LC3 to an amino group of phosphatidylethanolamine (PE) 44. The lipidation of LC3 is crucial for both the expansion and potential closure of the phagophore and serves as a late marker of autophagosomes. Moreover, Nath et al. proposed that ATG3 structure is adapted to function at highly curved membranes 45. Furthermore, ATG3 catalytic site is rearranged, enhancing its E2 conjugase activity, by the interaction with the intermediate conjugate ATG12-ATG5 46. The PIK3C3-C1 aids LC3 lipidation by utilizing the amphipathic lipid packing sensor (ALPS) motif of ATG14, which targets membrane curvature during the process 47. In addition, ATG8 family, both LC3 and GABARAPs, are important mediators of selective autophagy and vesicle trafficking (reviewed below).

The elongation of the phagophore requires a significant supply of lipids to sustain its growth. Although the intricate details of this process are not yet fully elucidated, it is widely acknowledged that it involves inputs from almost all intracellular compartments 34. Three potential mechanisms have been proposed to facilitate this lipid supply: vesicle-mediated delivery, direct extrusion from preexisting organelles, and direct protein-mediated lipid transport 48. These three mechanisms are likely interconnected and may act in a coordinated manner to ensure a continuous supply of lipids during autophagosome elongation.

The first mechanism involves vesicle-mediated delivery, where vesicles derived from various cellular organelles, such as the ER, Golgi apparatus, and endosomes, transport lipids to the phagophore membrane. ATG9 is the only transmembrane member of the ATG family. ATG9 vesicles are involved in transporting membrane materials for phagophore expansion, mainly in the first stages (seeds of membrane formation) 49. Notably, ATG9 harbors scramblase activity, essential for membrane growth, and it forms associations with ATG16L1 in endosomes, being mobilized from the Golgi-endosomal complex through the TRAPPIII (trafficking protein particle III) complex.^50^ However, ATG9 is not found in the autophagosome and it is thought to recycle back to its original location after contributing its membrane content to the growing autophagosome 51. In the case of yeast, ATG13 HORMA domain recruits ATG9 during autophagosome formation 52. Lastly, ATG9 vesicles have been proposed to establish membrane contact sites with lipid donor compartments, further contributing to the process 53. Coat protein complex II (COPII) vesicles have ben also reported to contribute to the phagophore elongation 54-56.

The second mechanism suggests a direct extrusion of lipids from preexisting organelles to the phagophore. This idea is supported by observations of certain autophagosomes forming in locations other than the ER, such as mitochondria or early recycling endosomes 42,57. Another possibility is the direct extrusion of phagophore from the ER 58. This direct transfer of lipids ensures a rapid and efficient supply of essential membrane components.

Lastly, the third proposed mechanism involves direct protein-mediated lipid transport. In this case, the best characterized is the ATG2 protein, which transfers tens of lipids, such as glycerophospholipids, between the ER and the phagophore in expansion 59. WIPI4 forms a complex with ATG2 to act as a tethering factor supporting the phagophore elongation 60,61.

#### Closure

Once the phagophore has expanded, its edges come closer together. The initial membrane structure undergoes transformation into a complete, sealed double-membrane autophagosome. This requires the scission of the inner and outer membranes of the phagophore, that become separated entities 62. Some key regulators of this crucial step are endosomal sorting complexes required for transport (ESCRT) machinery, ATG2-5, Rab GTPases and soluble N-ethylmaleimide sensitive factor attachment protein receptors (SNAREs) 63. Lastly, the closure of the phagophore requires phospholipid metabolism, specifically the synthesis of phosphatidylcholine 64.

#### Maturation

The maturation of the autophagosome is a sequential process that occurs after the phagophore closure and culminates in the formation of the autolysosome. Throughout this maturation, the autophagosome undergoes various transformations and interacts with endolysosomal compartments 65. Initially, the autophagosome fuses with early endocytic vesicles, early endosomes, multivesicular bodies, and late endosomes/lysosomes, leading to the formation of amphisomes. These amphisomes contain a mixture of both autophagic and endocytic materials 66. Subsequently, the autophagosome further matures and fuses with lysosomes, transforming into autolysosomes. In autolysosomes, a distinct lysosome-specific content, including hydrolases, combines with the autophagic material, facilitating the degradation of cargo and recycling of cellular components 67.

#### Fusion

After the formation of the autophagosome, it undergoes fusion with lysosomes, autophagolysosome, to release its contents for degradation. First, autophagosomes must move to lysosomes, usually at the perinuclear space, in a microtubule- and dynein-dynactin motor complex-dependent manner 68. Membrane-tethering factors have to be recruited to bring into close proximity both compartments, connecting the opposing membranes and/or promoting the formation of SNARE complexes, such as the heterohexameric complex HOPS (homotypic fusion and protein sorting), PLEKHM1 or TECPR1 67. HOPS complex is the best-studied and mediates autophagosome-lysosome fusion through interaction with syntaxin 17 (STX17) 69. Following tethering, the SNARE proteins present on the autophagosomal membrane, such as syntaxin 17, interact with SNAREs on the lysosomal membrane, including VAMP8 (vesicle-associated membrane protein 8) and SNAP29 (synaptosomal-associated protein 29) 70,71. These SNARE interactions drive the fusion of the two membranes, bringing the contents of the autophagosome into the lysosomal lumen 72.

Another critical player in the fusion process is the small GTPase protein RAB7, which regulates late endosome-lysosome and autophagosome-lysosome fusion. RAB7 promotes the movement and docking of lysosomes to autophagosomes, interacting with FYVE and coiled-coil domain-containing protein 1 (FYCO1) and Rab-interacting lysosomal protein (RILP), and the fusion of the membranes, by the collaboration with HOPS complex and SNARES 73,74. Finally, PI3KC3-C2 contains the UV-irradiation resistance-associated gene (UVRAG) subunit, instead of ATG14, and is a critical regulator of the last steps of autophagy. UVRAG recruits RAB7 and HOPS complex, mediating the fusion 75,76.

#### Degradation and autophagic lysosome reformation

Once the autophagolysosome has formed, it initiates the lysosomal degradation of autophagic materials. Initially, yeast Atg15 (or its unidentified mammalian counterpart) degrades the inner autophagosomal membrane, while leaving the outer membrane intact 77. This may due to the acquisition of different properties at the scission step of both membranes. Subsequently, a multitude of over 60 lysosomal hydrolases effectively digest the autophagic material to molecular level, functioning optimally under acidic pH conditions 78. It is well accepted that catabolites are transported to the cytoplasm via lysosomal transporters, decreasing autophagolysosome volume, and subsequently recycled by the cell to synthesize new molecules, structures, or organelles.

**Figure 2:**
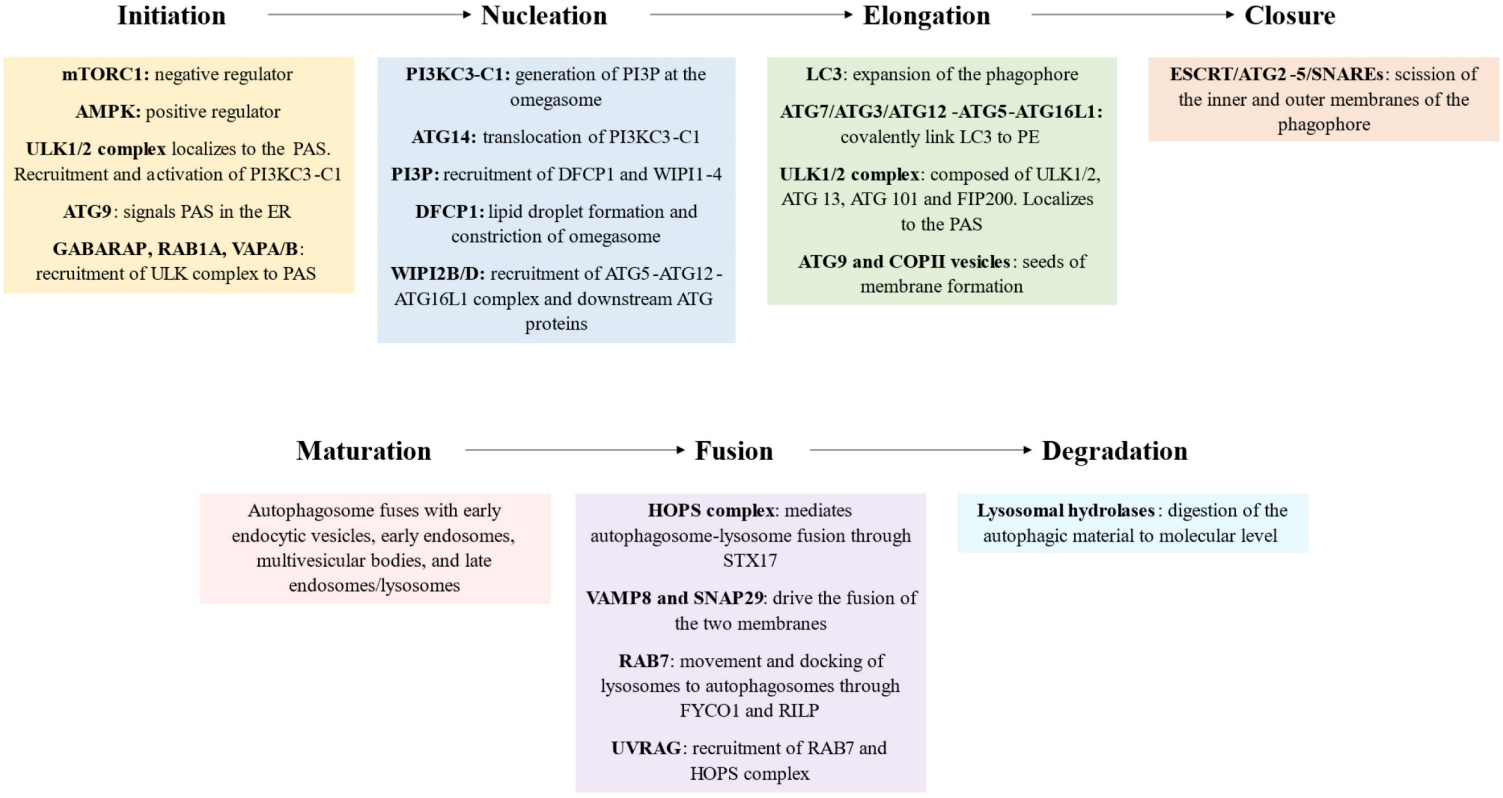
Schematic representation of the key molecular players of macroautophagy, steps and role. mTORC1: mammalian target of rapamycin complex 1, AMP-activated protein kinase: AMPK, ULK1/2: unc-51 like autophagy activating kinase 1/2, FIP200: focal adhesion kinase (FAK)-interacting protein of 200 kDa, PAS: phagophore assembly site, ER: endoplasmic reticulum, GABARAP: gamma-aminobutyric acid receptor-associated protein, VAMPA/B: VAMP-associated protein A/B, PI3KC3-C1: class III phosphatidylinositol 3-kinase complex I, PI3P: phosphatidylinositol 3-phosphate, DFCP1: double FYVE containing protein 1, WIPI1-4: WD-repeat protein interacting with phosphoinositides 1-4, LC3: microtubule-associated protein 1 light chain 3, PE: phosphatidylethanolamine, COPII: coat protein complex II, ESCRT: endosomal sorting complexes required for transport, SNAREs: soluble N-ethylmaleimide sensitive factor attachment protein receptors, HOPS: homotypic fusion and protein sorting, STX17: syntaxin 17, VAMP8: vesicle-associated membrane protein 8, SNAP29: synaptosomal-associated protein 29, FYCO1: FYVE and coiled-coil domain-containing protein 1, RILP: Rab-interacting lysosomal protein, UVRAG: UV-irradiation resistance-associated gene.

Autophagic lysosome reformation (ALR) is the final step of autophagy that restores the level of free lysosomes to maintain lysosome homeostasis 79. It initiates upon mTOR reactivation, a negative feedback to avoid excess of autophagy. During ALR, lysosomal membrane components assemble into tubular structures that protrude from autophagolysosomes. Subsequently, small vesicles, called proto-lysosomes, bud from these tubular structures 80. In their initial state, proto-lysosomes are pH-neutral and lack lysosomal luminal proteins. As they mature, they acquire acidity and incorporate lysosomal luminal proteins, transforming into nascent lysosomes 81. Overall, the main molecular players of macroautophagy are summarized in Figure 2.

#### Selective vs non-selective autophagy

Autophagy can be categorized into non-selective (bulk autophagy) or selective processes based on the material being degraded. Non-selective autophagy involves the random engulfment of various cytoplasmic contents, triggered by general stress conditions like nutrient deprivation, with the derived catabolites supporting cell survival 82. On the other hand, selective autophagy serves as cellular quality control, targeting and degrading specific components such as damaged organelles, protein aggregates, and invading pathogens. This process relies on specific autophagy receptors or adaptors that recognize cargo and interact with the autophagosome machinery 83,84. The cargo is then sequestered into autophagosomes for degradation in lysosomes. Selective autophagy has been found to target various cellular components, including aggregated proteins (aggrephagy), mitochondria (mitophagy), peroxisomes (pexophagy), ribosomes (ribophagy), endoplasmic reticulum (reticulophagy), lipid droplets (lipophagy), glycogen (glycophagy) and pathogens (xenophagy). Autophagy receptors, such as p62, optineurin, NDP52, or NBR1, play a crucial role in the autophagy process. These receptors have the unique ability to simultaneously bind to both the cargo that needs to be degraded and the autophagy machinery components, like LC3 or FIP200 85,86. Defects in the PINK1 and Parkin signaling in mitophagy are involved in the development of Parkinson´s disease 87,88.

#### Alternative autophagy

Alternative autophagy is a recently discovered form of autophagy that exhibits distinct characteristics from the canonical pathway, with the autophagosome membranes derived from the trans-Golgi membrane and its biogenesis independent of ATG5 and ATG7 89. Despite lacking these essential autophagy-related proteins, this alternative pathway is still capable of selectively degrading specific cellular cargo. Golgi stressors like etoposide, amphotericin B1, or genotoxic stress can induce alternative autophagy 90. Research into various diseases, including cardiovascular diseases, neurodegenerative disorders, cancer development (oncogenesis), and inflammatory bowel disease (IBD), has highlighted the significant role of alternative autophagy in their pathophysiological processes, offering new opportunities for investigating autophagy regulation complexities 91.

#### Microautophagy

Microautophagy is a distinct and less well-understood form of autophagy that involves the direct engulfment of cytoplasmic components by the lysosome or late endosome. This process allows for the selective degradation of specific cytoplasmic components, including organelles and protein aggregates, and plays a crucial role in cellular homeostasis and quality control 92. The lysosomal or endosomal membrane undergoes invagination, forming a tubular or vesicular structure protruding into the cytoplasm (Figure 1b).

Regarding the molecular machinery, endosomal microautophagy relies on multiple endosomal sorting complexes required for transport (ESCRT) systems 93. However, it is possibly the lest studied form of autophagy. Microautophagy can be also a selective process, e.g.: micro-ER-phagy through SEC62 receptor or proteins with the pentapeptide motif KFERQ via chaperone HSC70 94.

Microautophagy can be further categorized into three main types based on the engulfment mechanism: type 1, microautophagy with lysosomal protrusion, type 2, with lysosomal invagination; and type 3, with endosomal invagination 95.

### Chaperone-mediated autophagy (CMA)

Chaperone-mediated autophagy (CMA) is a selective form of autophagy that specifically targets and degrades individual cytosolic proteins (Figure 1C). Unlike macroautophagy, CMA directly delivers substrates to lysosomes for degradation 96. This highly regulated process plays a crucial role in maintaining cellular protein quality control, responding to cellular stress, and providing an alternative energy source during nutrient deprivation 97.

The hallmark of CMA is the recognition and targeting of substrate proteins by chaperone proteins. One of the key chaperones involved in CMA is HSC70, which selectively recognizes the pentapeptide motif KFERQ in the substrate proteins. Upon binding, HSC70 escorts the target protein to the lysosomal membrane, where it interacts with the lysosome-associated membrane protein type 2A (LAMP2A), a lysosomal membrane protein that forms a translocation complex that recognizes HSC70-substrate complexes 98,99. The substrate is then unfolded and translocated across the lysosomal membrane into the lysosomal lumen for degradation by lysosomal proteases. Overall, Figure 1 summarizes the main types of autophagy and their different steps.

## Biological relevance of autophagy. An overview

Autophagy is an essential factor related to several physiological processes, equally representing a pivotal pathophysiological mechanism of a broad spectrum of diseases 13. López-Otín & Kroemer described the hallmarks of health, a set of biological mechanisms that aid to understand health not only as the absence of pathology but also as a set of organizational and dynamic features that collectively maintain normal physiology 2. They divided the hallmarks of health into three major dimensions: spatial compartmentalization, sustainment of homeostasis over time, and adequate responses to stress. In this picture, autophagy is known to represent a central process implicated in adequate recycling and turnover of dysfunctional molecules and organelles that aid to keep homeostasis in the different cells and tissues. Thus, autophagy acts as an important cytoprotective mechanism implicated in the maintenance of cellular energy levels, components, and metabolites, as well as the elimination of cellular molecular damage 100. However, autophagy also orchestrates an array of biological processes, playing a dual and controversial role in different cellular events, aiding to understand its relevance in health and disease 13. In this section, a brief summary of the role of autophagy in different biological processes will be performed.

### Autophagy in the cellular response to stress

Autophagy is mostly considered a cellular stress response. Different environmental stressors are responsible for modulating the autophagy response such as nutrient deprivation, oxidative stress, hypoxia, endoplasmic reticulum stress, inflammation, intracellular pathogens, protein aggregates, toxic molecules and certain pharmacological agents 8,101. As a part of the cellular machinery implicated in stress responses, autophagy is induced by transcriptional and posttranscriptional reprogramming that leads to the activation of the autophagy proteins. Different transcriptional factors like MIT/TFE, PPARα, TFEB, ATF4, E2F1, C/EBPβ, FOXO, NF-κB, E93, STAT, and p53 are critically involved in the activation of autophagy in response to stress, whereas a broad spectrum of non-coding RNAs involved in the regulation of the Atg gene and other epigenetic mechanisms like histone modifications and DNA methylation can also participate in the activation of autophagy 102,103.

Autophagy is also integrated and related to several cellular processes like the cell cycle, apoptosis, and other processes 8. Under stressful conditions, compelling evidence supports that autophagy is able to induce and possibly execute cell cycle arrest programs, delimiting cell division and growth 104. This relationship can have important consequences for certain pathologies like cancer. Likewise, the relationship between autophagy and apoptosis is complex and frequently controversial. Both processes can be activated simultaneously by various stress pathways 105. Despite autophagy commonly blocks the induction of apoptosis, and apoptosis prevents the autophagic process, autophagy and apoptosis can appear concomitantly 106. From an evolutive perspective, autophagy is evolutionarily a more ancient event than apoptosis, and as both processes controls cell death and survival, they need to be narrowly linked 107. As autophagy has both pro-survival and pro-death functions, the cellular fate will depend on the extent of stress or damage and what the cell is capable of tolerating, as well as the functioning of the apoptosis process and its interplay with autophagy. Indeed, loss of autophagy and apoptosis function and communication is associated with different pathologies like cancer, neurodegenerative or cardiovascular diseases amongst others 107-109.

The relevance of autophagy as a cellular stress response can be observed in different regions of the body. Among various cell types, nervous system cells, especially postmitotic neurons, are remarkably susceptible to an array of insults, making autophagy a crucial stress response mechanism that safeguards the well-being and survival of these vulnerable cells 110. Conversely, prolonged exposure to stress can lead to a disruption in the autophagy control, leading to a defective or exacerbated autophagy, which is associated with a range of human diseases 111.

### Autophagy and aging

One of the most widely known mechanisms by which autophagy influences health and disease is through its tight association with aging. Several animal models have found that maintenance of proper autophagic activity is associated with extended longevity 112. Conversely, disabled autophagy is considered a primary hallmark of aging, also leading to unrestrained inflammasome activation, cellular damage, and oncogenesis, among other consequences 113. It should be noticed that both a reduction and an exacerbated autophagy are tightly associated with pathological aging and aging-related diseases. Previous works have found that autophagy is a process that seems to decrease with age, potentially contributing to the accumulation of damaged organelles and macromolecules, metabolic alterations in the cells and organelles, an increase in endoplasmic reticulum stress or decreased lysosomal proteolytic activity, among other consequences 112,114. On the other hand, uncontrolled autophagy might accelerate aging by increasing the number of senescent cells, cellular fast muscle fibers atrophy, cardiac hypertrophy, molecular and metabolic dysregulation, sarcopenia, neurodegeneration and other detrimental effects 114. Thus, balanced autophagy is essential to promote healthy aging and different strategies aimed to modulate this process are growingly being considered to ameliorate pathological aging and aging-related diseases 112.

### Autophagy and the immune system

Simultaneously, there is also a close relationship between the immune system and autophagy. Compelling evidence has found that autophagy is directly involved in the modulation of immune responses mainly by influencing the homeostasis, survival, activation, proliferation, differentiation, and cytokines production, of innate and adaptative immune cells including T and B lymphocytes, natural killer (NK) cells, macrophages, and dendritic cells (DCs) 115. Simultaneously, immunoglobulins, immune-related cells, and cytokines are directly involved in autophagy regulation both by inducing this process through specific products such as transforming growth factor (TGF)-β, interferon (IFN)-γ, interleukin (IL)-1, IL-2, and IL-12 or by inhibiting this process (IL-4, IL-10, and IL-13).^116^ Thus, autophagy is a process which influences and is regulated by acute and chronic inflammatory conditions, also collaborating with the immune responses to infections and other systemic challenges 117,118. In this context, previous works have identified perturbations in the autophagy process as a critical pathogenic mechanism of immune and inflammatory-related maladies like infections, autoimmunity, metabolic disorders, inflammatory bowel disease, neurodegeneration, cardiovascular diseases and cancer 117,119. Therefore, autophagy modulation is also a major target for prevention and therapy of immune-related diseases, although the knowledge of the mechanistic processes and role of autophagy in the context of inflammation requires additional efforts 120-122.

### Autophagy, metabolic and endocrine system

Autophagy has also been linked to metabolic regulation by different studies. Previous works differentiate between a metabolic and a quality control autophagy 123. The latter selectively eliminates a diverse range of cytoplasmic targets whereas the former is a response to starvation. As aforementioned, nutrient deprivation is a pivotal inductor of autophagy through the activation of AMPK and suppression of mTOR 124. Amino acids, fatty acids and glucose narrowly influences the core components of the autophagy machinery, controlling energy metabolism by tissue-specific effects and globally (endocrine effects) 125. In case of the former, nutrient deprivation and starvation are major inductors of autophagy in liver and heart, generating fatty acids and amino acids catabolized to obtain energy 126. In the liver, autophagy is linked to the processes of ketogenesis and gluconeogenesis, leading to the release of ketone bodies and glucose to feed the brain. However, as starvation prolongs, degradation and autophagy of adipose and muscle tissue is required to supply substrates to the liver, demonstrating that autophagy is a central mechanism implicated in the modulation of the metabolism in the body 126. In the endocrine system, autophagy orchestrates intracellular hormone levels both in peptide-secreting cells (targeting the secretory granules to control the levels of stored hormone) as well as in steroid secreting cells (targeting steroid-producing organelles) 127. As starvation favorably modulates autophagy, overnutrition can drive to an important dysregulation of autophagy, leading to the onset of a broad spectrum of disorders with a metabolic and endocrine base 125. For example, different conditions like sarcopenic obesity, insulin resistance and type 2 diabetes mellitus (T2DM) are characterized by an impaired autophagy related to a metabolic derangement, enhanced intracellular stresses and accelerated ageing in specific organs like the liver, pancreas, skeletal muscle and adipose tissue 128.

### Autophagy and tissue differentiation

During ontogenesis across diverse organisms, autophagy orchestrates a multitude of cellular processes, encompassing survival in nutrient-deprived conditions, programmed cell death (apoptosis), phagocytic events, organelle clearance, and miRNA regulatory functions 129. By employing animal models, pivotal mechanistic insights pertaining to autophagy regulation and its involvement in pathological states have been unveiled. Embryonic studies have unveiled specific autophagy-associated molecules responsible for the elimination of paternally inherited mitochondria and have identified autophagic components responsible for the clearance of protein aggregates during development 129. It has become evident that autophagy is a crucial process for maintaining the equilibrium and functionality of various tissues, influencing their development, differentiation, and capacity to undergo remodeling in response to stimuli or during periods of stress 130.

Autophagy, as an essential recycling mechanism, profoundly influences the destiny of various types of stem cells. Its role can be attributed to its significant contribution in maintaining the balance of a wide range of peptides and proteins, including growth factors, transcription factors, and crucial enzymes that play pivotal roles in cell functions such as proliferation, differentiation, and aging 131,132.

Moreover, autophagy exerts an impact on cell fate determinations by modulating mitochondrial content, energy generation, and epigenetic programming. Notably, in the context of aging and degenerative disorders, where the regenerative capacity of stem cells declines, autophagy assumes pivotal roles in shielding stem cells from cellular stress, emerging as a promising target in the field of regenerative medicine 133.

### Autophagy in diseases

**Figure 3:**
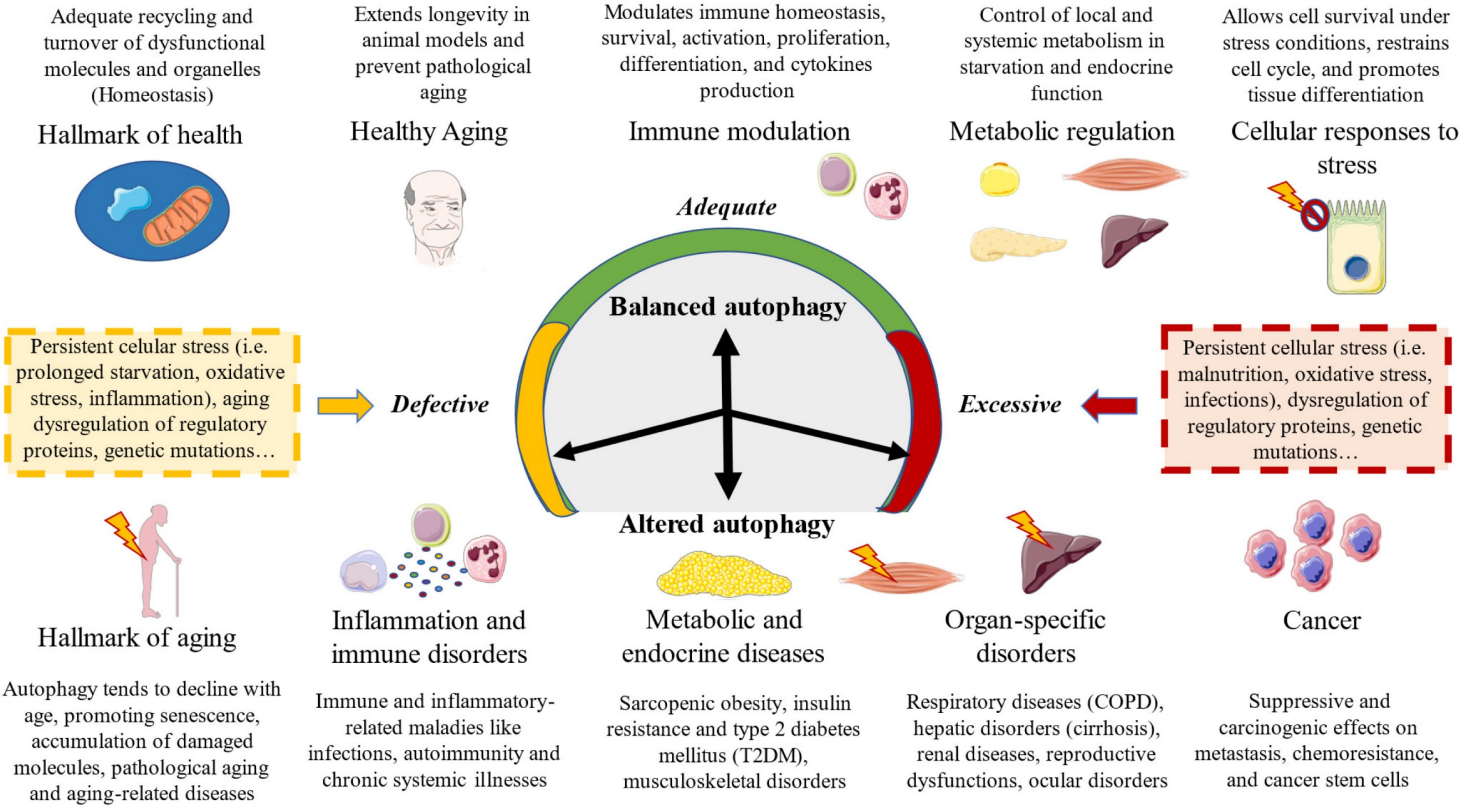
A summarized view of the biological relevance of autophagy in balanced and disrupted autophagy.

Autophagy is a relevant biological process in almost all systems and regions of the body. The relationship between autophagy and disease has been explored in previous works. Autophagy can act either as a protective or as a promoter mechanism of different maladies. A balanced autophagy is associated with protective mechanisms and health effects, whereas a disturbed autophagy either by defect or excess is a major trigger of disease (Figure 3). There are many underlying causes of an aberrant autophagy, particularly a prolonged stress exposure, dysregulation of autophagy proteins and specific genetic mutations. In case of the latter, the relationship between genes and impaired autophagy is receiving growing attention in the last years, as compelling evidence is revealing direct links between genetic defects of core autophagy genes, autophagy-associated genes, and genes encoding autophagic receptors with multiple diseases 134. In this sense, there are a group of pathologies known as congenital disorders of autophagy, which are identified by monogenic mutations and include EPG5-related Vici syndrome, beta-propeller protein-associated neurodegeneration due to mutations in WDR45, SNX14-associated autosomal-recessive cerebellar ataxia and intellectual disability syndrome, and three forms of hereditary spastic paraplegia, SPG11, SPG15 and SPG49 caused by SPG11, ZFYVE26 and TECPR2 mutations, respectively 135.

Besides these specific conditions and as aforementioned, autophagy plays a key role in the regulation of multiple processes like aging, immune modulation, metabolic and endocrine function, and an altered autophagy is associated with pathological aging, immune dysfunction and inflammation as well as several endocrine and metabolic diseases. But an impaired autophagy is observed not only in these conditions, but also in virtually all organ-specific and systemic disorders 3. For instance, autophagy is considered a critical process in heart and cardiovascular health, and an aberrant autophagy is associated with molecular, cellular, structural and functional impairments in the cardiovascular system 136,137. Because of that, autophagy has been recognized as a major therapeutic target for different cardiovascular diseases, aiming to stimulate (ischemia/reperfussion, cardiac lysosomal storage disease, diabetic cardiomyopathy) or to ameliorate this process (left ventricular hypertrophy; heart failure with reduced ejection fraction; anthracycline cardiotoxicity) 138. In the same line, neurodegenerative (Parkinson's, Alzheimer's or Huntington's disease) and psychiatric disorders (major depressive disorder, bipolar disorders or schizophrenia) are also characterized by an impaired autophagy in the nervous system, having been identified as a promising translational target for these conditions 139.

The role of autophagy in cancer has also been deeply explored by prior works. The evidence seems to support that autophagy acts as a tumor suppressor and promotor, with multiple effects on metastasis, chemoresistance and cancer stem cells 140. While certain autophagy modulators like rapamycin and chloroquine are employed in anticancer therapy to regulate autophagy, a comprehensive understanding of the precise mechanisms underlying autophagy's involvement in cancer is imperative and necessitates further investigation 141. As the complexities of autophagy in cancer biology unfold, continued research efforts are warranted to optimize its therapeutic potential and exploit its dualistic nature in a targeted manner for effective cancer treatments.

Finally, abnormalities in autophagy has been demonstrated in respiratory diseases like chronic obstructive pulmonary disease (COPD), hepatic disorders like cirrhosis, renal diseases, reproductive dysfunctions, ocular disorders, and in general, the different diseases related to aging, immune and metabolic dysfunction 3. In summary, autophagy plays a crucial role in determining human health and offers potential interventions to prevent or alleviate common illnesses. Preclinical evidence suggests that autophagy defects are particularly detrimental to post-mitotic cells, and that autophagy defects in healthy cells are typically associated with diseases due to disruptions in cellular homeostasis rather than a failure to adapt to nutrient scarcity, whereas cancer cells exploit autophagy whereas cancer cells exploit autophagy to cope with intracellular stress 142.

## Modulation of autophagy

Studying the modulation of autophagy, through both pharmacological and non-pharmacological approaches, is essential for understanding its role in maintaining cellular health and its involvement in various diseases. By identifying drugs and lifestyle interventions that can enhance or inhibit autophagy, researchers can develop targeted therapies for conditions like neurodegenerative disorders, cancer, and metabolic diseases. Autophagy modulation also offers insights into cellular stress responses, aging, and personalized medicine. Ultimately, this research holds promise for improving disease treatment, drug discovery, and promoting healthier aging and longevity.

### Pharmacological modulation

As discussed earlier, autophagy is a crucial process that significantly influences both health and disease. This opens up a wide range of therapeutic opportunities aimed at targeting and regulating autophagic flux within cells. Generally, promoting autophagy can lead to cell survival, while inhibiting it can result in cell death due to the buildup of toxic products 143. However, more research is needed in order to achieve better results and to know the possible side effects of the agents.

Pharmacological modulation of autophagy can involve both activation and inhibition of the autophagy process, depending on the specific aims and contexts. Activators of autophagy promote the initiation and progression of autophagosome formation, while inhibitors prevent the degradation of autophagosomes or disrupt the fusion of autophagosomes with lysosomes 144. In Table 1 we present a collection of the best-described autophagic activators and inhibitors.

Autophagy activators can either promote cell survival in the case of diseases characterized by accumulation of protein aggregates, such as neurodegenerative diseases or haemochromatosis, or lead to cell death when induces excessive autophagy, such as cancer cells 145. The main targets of this group are the inhibition of mTOR and activation of AMPK, the upstream regulators of macroautophagy. It is the case of rapamycin and rapalogs, arsenic trioxide or epigallocatechin-3-gallate (EGCG). A special group are the nutraceuticals, which achieve the activation of autophagy by pleiotropic pathways, e.g.: EGCG, polygonatum cyrtonema lectin, spermidine, resveratrol, allicin, ginsenosides and curcumin.

On the other hand, autophagy inhibitors are a suitable approach for cancer therapy due to the protective role of autophagy in cancer 146,147. Autophagy inhibitors can target various steps of the autophagy process. For instance, wortmannin or 3-methyladenine act on the nucleation step by targeting PI3KC3, while pepstatin-A and bafilomycin A1 affect lysosomes, either by inhibiting lysosomal acidification or by hindering the fusion with autophagosomes.

### Lifestyle Medicine and Autophagy

Lifestyle medicine is a growing area of research based on the modulation of key health behaviors such as diet, physical activity, sleep, and environmental factors like tobacco, social relationships, and also stress management 190-192. Lifestyle medicine is increasingly being considered a crucial mechanism to modulate autophagy, aiding to explain the multiple benefits from this type of approaches for health and also for the management of chronic diseases 193,194. In this section we will summarize the relevance and implicated mechanisms of different lifestyle factors on autophagy, exploring the role of diet (considering nutrients, foods and dietary strategies), physical activity, sleep and circadian regulation, tobacco, alcohol, air pollution, sunlight exposure and psychosocial stress. Table 2 summarizes the main findings of this topic.

#### Diet

Diet is a major modulator of autophagy. More specifically, available literature distinguishes between specific nutrients present in certain groups of foods or in the form of supplementation and specific dietary strategies such as caloric restriction and intermittent fasting 195,196. All these components can directly act through the biochemical regulation of different products such as AMPK, SIRT-1, eIF5A, or GCN2, ultimately leading to the modulation of the main proteins implicated in the autophagy process like ULK1, TFEB, FOXO1, ATF-4 or CHOP 195.

Firstly, there are many nutrients with significant health/translational properties (nutraceuticals) that have been demonstrated to modulate the process of autophagy. To provide some examples, amino acids (i.e., leucine), fatty acids (i.e., omega 3 polyunsaturated fatty acids), vitamins (carotenoids and retinoids, ascorbic acid, calciferol, tocopherols, and tocotrienols), coenzyme Q10, bioactive compounds (i.e., mainly polyphenols like curcumin, caffeine, EGCG resveratrol, allicin), minerals like zinc or iron, ergothioneine, lipoic acid, N acetylcysteine and spermidine.^197^-^201^ Some of the most relevant nutraceuticals with a role in the modulation of autophagy, targets, and biological mechanisms appear summarized in Table 1. Not only isolated nutrients but also those contained in foods are important modulators of autophagy. Plant foods are perhaps the major modulators of these processes, especially regarding their content in a broad spectrum of nutrients involved in the regulation of this process, such as vitamins and several bioactive compounds 195,202. After plants, foods like fish and seafood, eggs, and in general, different groups of food commonly consumed in healthy dietary patterns such as Mediterranean Diet (Med Diet) have also significant effects on upregulating autophagy 203,204. Generally, healthy, non-processed or minimally processed foods and dietary patterns tend to stimulate the process of autophagy, thus explaining their anti-aging, anti-inflammatory, and beneficial health outcomes 205. Conversely, unhealthy foods and dietary patterns such as westernized diets, abundant in refined sugar, unhealthy fats, ultra-processed foods, salt and controversial additives are mostly related to an inhibition in the autophagy process 206,207. Thus, dietary patterns, foods, and nutrients are major modulators of autophagy, aiding not only to preserve health but also representing promising translational approaches for several diseases 136,164,197,208,209.

Additionally, not only the quality and type of nutrients and foods ingested but also the quantity and timing of intake seem to be relevant for modulating autophagy. Thus, calorie restriction (CR) and intermittent fasting are both strategies tightly linked to the activation of this process. CR is a type of nutritional intervention of reduced energy intake but supported with an adequate nutrition, representing a valuable clinical strategy in the medical management of obesity, cancer and cardiometabolic disorders 210. Besides, CR has also been related to anti-aging effects, being its stimulatory role in autophagy defined as a critical mechanism explaining this interplay 211,212. Intermittent fasting could be defined as the complete deprivation of food but not water, with intervening periods of normal food intake 213. Their stimulatory effects on autophagy are similar to those observed in CR, although in comparison it is commonly associated with greater adherence rates than CR 214. Indeed, it is common that intermittent fasting entails a reduced caloric intake by limiting the number of daily intakes, explaining the similar effects of both strategies. CR and intermittent fasting stimulates autophagy mainly by decreasing mTOR signaling by reducing insulin and IGF-1 levels and increasing the AMP/ATP ratio that leads to the activation of AMPK as well as several products involved in the stimulation of this process like ATG6, ATG7, ATG8, LC3-II, Beclin1, p62, Sirt1, LAMP2, ULK1 and ATG101 196,214. The favorable effects on autophagy of both CR and intermittent fasting seems to be involved in the increase of fat mobilization, oxidation, metabolic flexibility, insulin sensitivity and redox imbalance together with a reduction in systemic inflammation, cardiovascular risks and body weight 215. Hence, either CR or intermittent fasting are valuable and similar strategies to preserve health and aid in the clinical management of several diseases 216. Nonetheless, despite the heterogeneity observed in the available literature, some articles reflect that intermittent fasting and CR could potentially exert different effects and applications depending not only on adherence but also on the purposes of the intervention 217,218. Also, there are different strategies of intermittent fasting that could bring different health outcomes such as alternate-day fasting, other similar full-day fasting patterns, and time-restricted feeding 219. More studies are warranted to fully understand the precise relationship between CR and intermittent fasting with autophagy, especially to compare their effects on different tissues or in various types of fasting strategies 214,220.

Overall, diet could be considered a pivotal modulator of autophagy. Including specific types of nutrients and foods, commonly present in a healthy dietary pattern like Mediterranean diet favorably regulates this process, whereas refined and ultra-processed foods usually have the opposite effect. CR and intermittent fasting are two widely studied nutritional strategies that can also stimulate autophagy and exert significant anti-aging and translational applications, although further studies are required in healthy individuals and patients with different pathologies.

#### Physical activity

Physical activity (PA) is another major representant of lifestyle medicine with several benefits for sustaining health and a critical support for several chronic diseases 221. Physical inactivity or sedentary behavior emerges as a consequence of multiple factors that not only depend on the individual but also on society itself. According to the World Health Organization (WHO), physical inactivity is considered the fourth leading risk factor for global mortality 222. Thus, sedentary individuals face an increased risk of developing a wide spectrum of pathologies, ranging from cardiovascular diseases, metabolic disorders, cancer, osteoporosis, and other musculoskeletal issues, ultimately leading to a diminished quality of life 223. PA and exercise have multiple systemic effects on the body, leading to important adaptations and effects in the musculoskeletal, cardiovascular, nervous, metabolic and immune system, with a crosstalk between all these systems through different products (exerkines) 224. In this context, previous studies have found that PA plays a central role in the regulation of autophagy, potentially aiding to explain its multiple benefits on health and disease 225. Compelling evidence supports that PA increases several activators of the autophagy in the skeletal muscle like FOXO, TFEB, AMPK or ROS amongst others, promoting an increase of autophagy capacity and autophagy flux, eventually driving to the elimination of damaged organelles and proteins, improving mitochondrial function/oxidative capacity, regulating glucose, protein synthesis, muscle mass maintenance and exercise performance 226. Conversely, decreased activation of autophagy in the skeletal muscle is related to significant local and systemic consequences, driving to significant concerns like myopathies, muscle atrophy, exercise intolerance and insulin resistance 227. Not only the skeletal muscle, but also other regions of the body benefits from the positive effects of PA in the autophagy. The brain is one of the regions more favored by PA, as it regulates multiple processes and products essential for brain health and cognitive functions, aiding to prevent or improve the medical management of neurodegenerative and age-related disorders in the brain 228. Among the benefits of PA in the brain, it has been shown that exercise ameliorates autophagy and apoptosis dysfunction, leading to significant improvements in the affected patients 229. Also, an increased detection of autophagy markers like Beclin-1, ATG12, ATG16 and LAMP-2 is also observed in peripheral blood mononuclear cells (PBMCs) after 8 weeks of PA, along with a decreased apoptosis and inflammasome NLRP3 activation 230. Therefore, the association between PA and autophagy is overwhelmingly important, supporting the potential of considering PA as a non-pharmacological approach to favorably modulate autophagy in health and disease conditions.

On the other hand, different studies have found that the effects of PA on autophagy seems to be tissue- and exercise dependent on different exercise regimens to compare their precise role on this process. Pinto et al.^231^ compared the acute effects of endurance (END), exhaustive (ET), strength (ST), and concurrent (CC) physical exercise on various markers of autophagy in the gastrocnemius muscle, heart, and liver of mice. They observed that each type of PA presented different effects on autophagy markers in these regions, suggesting that each variant of exercise could be differentially receipted according to the patient´s features. In more detail, for gastrocnemious muscle samples the main alterations were observed after 6 hours of ST, whereas the markers of autophagy for the CC group were increased. The Beclin 1 and ATG5 levels in the heart were downregulated in the ET group. Sqstm1/p62, one of the autophagy markers, were shown to be increased in the cardiac tissue of the END and ST groups, whereas for the ET group, the levels of the liver protein ATG5 were downregulated. Simultaneously, a systematic review conducted by Chen et al.^232^ observed that acute ST was acutely associated with a decreased autophagy demonstrated by reduced levels of LC3-II and increased SQSTM1, although long-term ST was oppositely associated with an increased LC3-II and autophagy with a decrease in SQSTM1. They equally observed that other markers of autophagy like ULK1, Beclin-1, ATG12, BCL2 can also differentially change according to the type of exercise and in a tissue-dependent manner, and also that moderate and vigorous END was not associated with changes in the autophagy process, although other works have found a positive effect of this type of training on the autophagy 233,234. Likewise, trained subjects with a combination of ST with sprint exercises also presented enhanced autophagy defined as an increase in LC3-II and a decrease in p62 in comparison to other trained subjects that did not follow this protocol 235. Additionally, there are studies comparing the effects of high intensity interval training (HIIT) with moderate-intensity continuous training (MICT) on autophagy 236. Interestingly, they observed that both regimens were associated with enhanced autophagy 3 h post-exercise in skeletal muscle, although in PBMCs, the autophagy increased after HIIT and MICT in men, but not in women, suggesting a differential effect of both trainings depending on the tissue and sex.

Overall, PA exerts significant improvements in the autophagy that plays a pivotal role in the body adaptations to the exercise and also in several systemic processes essential for health and alleviation of various diseases. However, the type of training program, the considered tissue, sex and other factors are important to determine the precise effects of PA on autophagy. Future works should be performed to deeply characterize this intricate relationship.

#### Sleep and circadian regulation

Sleep fulfills several important functions for the homeostasis of the organism: restoration of energy levels, reparation of tissues, memory consolidation, maintenance of brain function, support of immune and endocrine system and psychological well-being 237-239. Numerous studies have demonstrated that sleep deprivation negatively affects health and quality of life, including cognitive functions such as mood, cognition, and memory 240-242. It has been demonstrated that basal autophagy is crucial for preventing the buildup of aberrant cytosolic proteins in neurons, whereas disruption of basal autophagy may result in neurodegeneration, as seen by a significant loss of neurons 243. In addition, autophagic activity follows the circadian rhythm 244,245. In *Drosophila*, sleep decreases autophagosome levels under unperturbed conditions and a strong and sustained downregulation of autophagosomes increases sleep 246. Also, in models expressing the mutant Huntingtin protein the induction of autophagy via overexpression of *Atg8a* (homolog of mammal LC3s and GABARAPs) achieves the reestablishment of normal sleep habits 247.

In mice, 5 days of sleep fragmentation led to the dysregulation of autophagy in specific brain regions, with the striatum and hippocampus showing heightened sensitivity. In the striatum, levels of BECN1, LC3-II, and p62 were increased, while in the hippocampus, LC3-II was elevated, but BECN1 and p62 were decreased 248. Interestingly, these changes had no impact on autophagy in the frontal cortex, indicating a region-specific response to sleep fragmentation. Another study showed that sleep-deprivation in rats also increases the expression of Beclin1, PINK1, parkin, p62, and LC3. Then, the treatment with propofol significantly reduces the levels of these proteins in hippocampal neurons, i.e., inhibits excessive autophagy, and could improve the impairment of learning and memory caused by sleep deprivation 249. Similar results are obtained in mice treated with modafinil, which also reduces excessive autophagy in hippocampus and likely activates P13K/Akt/mTOR/P70S6K signaling pathway 250. A study in rats revealed that sleep deprivation induces oxidative stress in the thyroid, which combined with alterations in autophagy- and apoptosis-related proteins, lead to an imbalance between autophagy and apoptosis in the organ 251. Consequently, an increased number of apoptotic cells were observed, suggesting potential damage to the thyroid. Finally, Kumar et al. performed a systematic review that indicate that autophagy dysfunction is connected to both acute and chronic neurodegenerative changes, as well as pathophysiological and behavioral alterations, all of which are linked to rapid eye movement (REM) sleep loss 252. Moreover, the increased levels of noradrenaline during REM sleep disruption may potentially affect autophagy in neurons, resulting in disturbances to neuronal integrity, homeostasis, and overall brain functions. These disturbances could ultimately play a role in the development of associated neurodegenerative disorders.

#### Tobacco

The tobacco epidemic is a significant global public health challenge. It is one of the most notoriously overused drugs among rural and urban populations in underdeveloped countries 253. The rate of tobacco product consumption and the number of smokers have both been steadily rising globally over the past ten years. As a result, the World Health Organization (WHO) estimates that almost 7,000,000 deaths are directly related to tobacco use 254.

*In vitro* exposure of bronchial epithelial cells and *in vivo* exposure of mice to cigarette smoke extracts (CSEs) and cigarette smoke, respectively, lead to defective autophagosome maturation, impairing the autophagic flux 255. These may occurr due to the observed accumulation of bicaudal D1, p62 and ubiquitin-associated p62 oligomers. Another study reveals a connection between tobacco smoking and impaired autophagy in alveolar macrophages, the cells responsible for clearing bacteria from the lungs. Smokers have an increased risk of lung infections, and the research shows that alveolar macrophages in smokers accumulate autophagosomes and p62, indicative of a dysfunctional autophagy process 256. This defect hinders the clearance of protein aggregates, impairs mitochondria function, and disrupts the delivery of bacteria to lysosomes. In addition, autophagy is increased in lung tissue from COPD patients. *In vitro* experiments using human pulmonary epithelial cells exposed to CSE showed a rapid induction of autophagy. The study identified the transcription factor Egr-1 as a critical factor promoting autophagy and apoptosis in response to cigarette smoke exposure 257. Lastly, CSE was found to impair autophagy in U937 macrophage-like cells, leading to the accumulation of galectin-8 and the autophagic adaptor protein NDP52, which is also observable in lung tissue and blood circulation of COPD patients. Soluble galectin-8 induced the release of interleukin-6 (IL-6) in bronchial epithelial cells via PI3Kα signaling 258. The study suggests that increased galectin-8 due to impaired autophagy induced by CSE may contribute to the development of COPD and could serve as a potential biomarker for the disease.

#### Alcohol

Drinking alcohol is linked to a variety of harmful medical effects that essentially affect every system in the body 259. Additionally, drinking alcohol has been linked to a number of cancers, which may be explained by the genotoxic effects of acetaldehyde, the generation of reactive oxygen and nitrogen species (ROS and RNS), modifications to the metabolism and DNA methylation of folate, impaired immune surveillance, nutritional deficiencies, and elevated estrogen levels 260.

In this sense, chronic alcohol consumption is a common risk factor for the onset of alcoholic liver disease (ALD). ROS and local inflammation are the main causes of alcohol-induced hepatocellular injury, which harms the structure of the liver and ultimately results in increased cell loss from the liver 261. There is not fully comprehension of how drinking alcohol affects the autophagic system of the liver. The studies suggest that alcohol can induce autophagy in the liver, acting as a cellular protective mechanism against acute ethanol-induced steatosis and liver injury 262,263. Ethanol metabolism through alcohol dehydrogenase (ADH) and CYP2E1 generates reactive metabolites that are required for autophagy induction. Ethanol-induced autophagy selectively removes damaged mitochondria and lipid droplets accumulated in liver cells with ethanol treatment, contributing to its beneficial effects in alcohol-induced liver disease 264. Pharmacological induction of autophagy by rapamycin or carbamazepine suppresses acute alcohol-induced steatosis and liver injury in mice 265. However, the effects of chronic ethanol exposure on autophagy are the inhibition of hepatocellular autophagy and induction of apoptotic cell death in Wistar rats 266. Nonetheless, enhancing hepatic autophagy seems to be beneficial in ALD, and specific enhancers of autophagy may hold promise as potential therapeutic interventions.

#### Air pollution

Air pollution primarily consists of particulate matter (PM) containing hazardous airborne particles suspended in the atmosphere. Studies have shown that air pollution can trigger autophagy in human lung epithelial cell lines, potentially leading to impairment in pulmonary function. The underlying mechanism involves oxidative stress induced by PM_2.5_ (particles with a diameter of less than 2.5 µm), which leads to accumulation of LC3 and elevated expression of BECN1 and ATG5, key components involved in autophagy 267. Conversely, inhibiting autophagy or depleting its resources can result in varied toxic effects, depending on the specific context 268. Mammalian cells rely on selective autophagy to effectively eliminate particulate matter, nanoparticles, toxic metals, and exposure to smoke without harming cytosolic components 269. One of the first organs exposed to PM is the skin. Results from Park et al. showed that PM contributes to skin aging by impairing collagen synthesis and triggering inflammation, along with an increase in autophagy which may indicate a potential reparative role in response to PM-induced stress 270.

#### Sunlight exposure

The primary risk factor for the development of skin cancer and skin photoaging is exposure to ultraviolet (UV) radiation from sunshine and indoor tanning beds. The most prevalent is UVA, which makes up around 95% of solar UV radiation and the remaining 5% of solar UV light is made up of UVB, and UVC is blocked by ozone. UV light is the primary source of vitamin D for humans 271.

UVA, UVB, and UVC stimulate the creation of autophagosomes and the overexpression of autophagy markers. It is believed to be a protective response against skin from ultraviolet radiation-driven photoaging or skin cancer 272,273. Autophagy degrades oxidized phospholipids and protein aggregates generated by UVA-induced ROS production, which are the best-known mechanism that activate autophagy.^274^ Deeply, oxidative stress seems to activate AMPK autophagy signaling 275.

On the one hand, UVA exposure leads to the up-regulation of p62, triggering a positive feedback loop with Nrf2 (nuclear factor (erythroid-derived 2)-like 2) to counteract oxidative stress. Moreover, p62 also forms a negative feedback loop with PTEN (phosphatase and tensin homologue deleted on chromosome 10), a tumor suppressor in melanoma cells, suggesting that p62 acts as an oncogene in UVA-associated melanoma development and progression 276. PTEN stimulates the autophagic flux by the inhibition of PI3K/AKT/mTOR signaling 277,278. On the other hand, the alterations in autophagy induced by UVA are a result of impaired autophagic flux caused by the inactivation of cathepsin B, which may play a role in UVA-induced skin photodamage 279.

#### Psychosocial stress

Alterations in autophagy have been linked to typical stress response pathways, and abnormal autophagy appears to be related to neuropsychiatric diseases 280. Studies conducted after death on people with major depressive disorder revealed deficiencies in the autophagy regulating mechanism mTOR. Additionally, it was discovered that antidepressants work through the mTOR pathway and reverse the psychological consequences of chronic stress 281.

Findings from the impact of abused substances indicate the vital role of autophagy in regulating emotional and cognitive behavior in response to psychological stress, particularly in the catecholamine synapse and neuronal protection 282. Autophagy plays a significant role in promoting adaptive emotional outcomes that contribute to overall wellbeing. However, in situations of chronic or persistent stress, where changes in neurotransmitter activity lead to maladaptive neuronal alterations, autophagy may be continuously impaired, leading to a gradual decline in synaptic function and ultimately to neurodegeneration 282. Indeed, chronic nicotine administration in mice exposed to mild chronic unpredictable stress, improved cognitive impairments and neuropathological alteration of dentate gyrus neurons caused by chronic stress in mice 283. The neuroprotective effects of nicotine were associated with an enhancement of the autophagy signaling pathway, involving the upregulation of autophagy markers (BECN1 and LC3 II). Paradoxically, these effects were also linked to the activation of the PI3K/Akt/mTOR signaling pathway.

On the other hand, epigenetic modifications play a crucial role in regulating autophagy and are linked to stress response and neuropsychiatric disorders. The HSP90 co-chaperone FKBP51 activates autophagy genes in response to antidepressants by reducing DNA methylation and increasing gene expression, including BDNF involved in neurogenesis 284. Stress conditions are associated with epigenetic changes that influence autophagic clearance of RNA from mobile genetic elements, while chronic stress may impact autophagy-related genes, potentially affecting neurogenesis 285. Autophagy's compensatory role in clearing aberrant transcripts may be crucial during chronic stress. Lastly, an interesting studio analyzed the relationship between psychosocial stress and intestinal autophagy in both intestinal bowel disease (IBD) patients and animal model. The findings suggest that psychosocial stress may exacerbate IBD by enhancing intestinal autophagy through gut microbiota modulation and inflammation, highlighting autophagy as a potential therapeutic target for psychosocial stress-related IBD 286.

## Conclusions

Autophagy is a growing area of research that has been related to several physiological and pathological processes, although their biological significance remains to be further explored. Compelling evidence define autophagy as a pivotal mechanism in the context of cellular stress response, aging, immunity, metabolism, tissue differentiation, and systemic illnesses like cancer, cardiovascular disease, or neurological disorders. Besides, different types of autophagy like macroautophagy, microautophagy, and chaperone-mediated autophagy are currently recognized by the available literature, each of them entailing a complex molecular background. Strategies involved in autophagy modulation include pharmacological interventions with potential clinical applications (Table 1). Besides, lifestyle medicine has also a notable role in the modulation of autophagy, as shown in Table 2. Diet, exercise, sleep habits, and environmental factors can all have a relevant influence on the process. The inclusion of lifestyle medicine in autophagy research creates new opportunities for individualized therapies that enhance health and longevity, although further efforts in this field are still warranted.

## Figures and Tables

**Table 1:** Pharmacological activators and inhibitors of autophagy. (*) Nutraceuticals able to modulate autophagy

**Pharmacological agent**	**Chemical structure**	**Source**	**Autophagy-associated targets**	**Mechanism of action**	**References**
**Activators**					
Rapamycin (Sirolimus)	Macrocyclic lactone	*Streptomyces hygroscopicus*	mTOR	Inhibits mTORC1 (acute and chronic exposure) Inhibits mTORC2 (chronic exposure) Rapamycin binds FKBP12 and act as an allosteric inhibitor	(148)
Temsirolimus	Derivative of rapamycin	Synthetic analog of rapamycin			(149)
Everolimus (RAD-001)	Derivative of rapamycin	Synthetic analog of rapamycin			(150)
Erlotinib	Quinazolinamine	Synthetic compound	EGFR	Inhibition of the EGFR signaling by interaction with ATP binding site	(151)
Imatinib	2-phenyl amino pyrimidine	Synthetic drug	BCR-ABL c-Abl Abl-related gene	Inhibits selectively tyrosine kinases Sequestering of the Bcr-Abl protein into autophagosomes	(152,153)
Dasatinib (BMS-354825)	Derivative of aminopyrimidine	Synthetic compound	Src/Abl family kinases	Reduces the phosphorylation of AKT, mTOR, p70S6K, and S6 kinase expression Requires BECN1 and ATG12	(154,155)
Vorinostat (SAHA)	Hydroxamic acid derivative	Synthetic compound	HDAC	Acetylation of ATG proteins leading to hyperactivation of PIK3C3 Upregulation of LC3 Inhibition of mTOR	(156-158)
Arsenic Trioxide	Amphoteric oxide	Processing of the mineral arsenic	TFEB ROS PI3K/AKT/mTOR	Nuclear translocation of TFEB Inhibition of PI3K/AKT/mTOR pathway Generation of ROS	(159)
Epigallocatechin-3-gallate*	Polyphenol*.*	Green tea leaves	mTOR-AMPK	Induces activation of AMPK and inhibition of mTOR	(160,161)
Polygonatum cyrtonema lectin*	Lectin	Rhizomes of* Polygonatum cyrtonema*	Mitochondria-mediated ROS-p38-p53 pathway	Accumulation of mitochondrial ROS, activating p38 and p53	(162,163)
Spermidine*	Polyamine	Dry soy bean, chicken liver, green peas, corn, shell fish, and blue cheese	Acetylation of histones	Modifies epigenetic landscape Increases the expression of ATG	(164,165)
Resveratrol*	Polyphenol	Grapes, apples, plums, blueberries, and peanut	mTOR, AMPK, SIRT1	Inhibiting mTOR via several routes, activates AMPK and SIRT1	(166-168)
Allicin*	Sulfoxide	Allium sativum	p53, mTOR and AMPK	Modulation of the pathways leading to autophagy	(169,170)
Ginsenosides*	Saponins	Ginseng root	Autophagy-related pathways	Modulation of the pathways leading to autophagy	(171,172)
Curcumin*	Diarylheptanoid	*Curcuma longa*	ROS mTOR	Inhibiition of mTOR Overexpression of autophagy-related proteins	(173-175)
**Pharmacological agent**	**Chemical structure**	**Source**	**Molecular target**	**Mechanism of action**	**References**
**Inhibitors**					
Wortmannin	Furanosteroid	*Penicillium funiculosum Talaromyces wortmannii*	PI3KC1 (transient) PI3KC3 (persistent)	Inhibition of the production of PIP3	(146)
3-Methyladenin	Adenin	*Saccharomyces cerevisiae*	PI3KC1 (persistent) PI3KC3 (transient)	Inhibition of PI3K Promotion of autophagic flux under nutrient-rich conditions (dual role)	(176,177)
Chloroquine	Aminoquinoline	*Cinchona officinalis*	Lysosomal pH SNAP29?	Slows down lysosomal acidification Inhibition of the fusion of autophagosomes with lysosomes	(178,179)
Pepstatin-A	Hexapeptide	*Streptomyces sp*	Cathepsin D and E	Inhibition of lysosomal proteases Accumulation of LC3-II	(146,180)
Bafilomycin A1	Macrolide	*Streptomyces sp*	Vacuolar-ATPase SERCA	Reduce lysosomal acidification Disrupt autophagosome-lysosome fusion	(181,182)
Hydroxy-chloroquine	Derivate from chloroquine	Synthetic modification of cloroquine	Lysosomal pH	Slows down lysosomal acidification Inhibition of the fusion of autophagosomes with lysosomes	(183,184)
Spautin-1	4-quinazolinamine	Synthetic compound	Ubiquitin-specific peptidase 10 and 13	Inhibits autophagy by the degradation of PI3K3 and BECN1	(185,186)
SAR405	Pyrimidine	Small molecule inhibitor	Class III PI3K	A selective ATP-competitive inhibitor of PI3K class III	(187)
siRNAs	20 to 24-bp double-stranded RNA	Synthetic compound	ATG mRNA	Knockdown the autophagy machinery, e.g.: ATG5	(188,189)

**Table 2:** Lifestyle habits and their influence on autophagy.

Lifestyle habit	Effectors	Influence on autophagy	Main outcomes of the autophagy	Reference
Mediterranean Diet	Fruits, vegetables, fish, rice, olive oil and eggs	Stimulation	Anti-aging, anti-inflammatory, improved cardiovascular health and enhanced brain function	203-205
Calorie restriction	Reduced energy intake. Adequate nutrition	Stimulation by decreasing mTOR signalling and activation of AMPK	Increase of fat mobilization, oxidation, metabolic flexibility, insulin sensitivity and redox imbalance Reduction in systemic inflammation, cardiovascular risks and body weight Anti-aging	196, 210-212, 214-220
Intermittent fasting	Periodic cycles of eating and fasting			196, 213-220
Physical activity	Increases FOXO, TFEB, AMPK or ROS levels and AMP/ATP ratio	Activate autophagy in skeletal muscle	Regulation of glucose, protein synthesis, muscle mass maintenance and exercise performance Attenuate aging-associated autophagic dysfunctions leading to neurodegeneration	225-234
Sleep deprivation	Autophagy machinery (BECN1, LC3 and p62)	Impairment of the levels of ATG proteins in hippocampus and striatum	Autophagy dysfunction is associated with neurodegenerative and behavioral alterations, all linked to REM sleep loss. Propofol and modafinil improve cognitive function loss by sleep deprivation in rodent models	243-252
Tobacco	Egr-1 Bicaudal D1, p62, galectin 8, NDP52	Induction of autophagy Defective autophagosome maturation	Accumulation of bicaudal D1, p62 and autophagosomes Development of COPD	255-258
Alcohol consumption	Ethanol metabolites	Stimulation (Acute) Inhibition (Chronic)	Chronic consumption leads to ALD. Autophagy is a protective response against acute ethanol induced steatosis and liver injury. Chronic consumption inhibits autophagy and induces apoptosis	262-266
Air pollution	Particulate matter	Stimulation	PM_2.5_-induced oxidative stress PM2.5 leads to accumulation of LC3 and overexpression of BECN1 and ATG5 Impairment in lung function Skin aging	267-269
Sunlight exposure	UVA-induced ROS	Stimulation or impairment	Skin photoaging and cancer	272-279
Psychosocial stress	BECN1, LC3II, FKBP51	Stimulation (acute) Impairment (chronic)	Promotes adaptive emotional outcomes (acute) Gradual decline in synaptic function and neurodegeneration (chronic) Exacerbate IBD, gut microbiota dysbiosis and inflammation Antidepressants reverse the psychological consequences of chronic stress via mTOR.	280-286
